# Draft genome sequences of *Psychrobacter* spp. isolated from Atlantic salmon (*Salmo salar*) survived winter cold stress in Atlantic Canada

**DOI:** 10.1128/mra.00971-25

**Published:** 2025-12-23

**Authors:** Xiaoji Liu, Steve Leadbeater, Mruthula Narayan, Skyler Umlah

**Affiliations:** 1 Agriculture and Agri-Food Canada, Lacombe Research and Development Centre 98668, Lacombe, Alberta, Canada; 2 Fisheries and Oceans Canada, St. Andrews Biological Station 6344, St. Andrews, New Brunswick, Canada; 3 Faculty of Science, University of British Columbia 8166, Vancouver, British Columbia, Canada; 4 Faculty of Science, Red Deer Polytechnic 6827 , Red Deer, Alberta, Canada; California State University San Marcos, San Marcos, California, USA

**Keywords:** Atlantic salmon, *Psychrobacter*, cold water disease

## Abstract

We present the draft genome sequences of 19 *Psychrobacter* spp. isolated from the skin of Atlantic salmon raised in flow-through systems in Atlantic Canada. All of these salmon survived winter cold stress with or without clinical symptoms (lesions).

## ANNOUNCEMENT

Cold-water diseases in Atlantic salmon (*Salmo salar*) caused by bacterial pathogens such as *Moritella viscosa* or *Tenacibaculum* spp. negatively impact salmon farm production (1–3). However, certain individuals within the population develop signs of disease, whereas others do not. To understand how skin commensal bacteria may protect salmon from developing lesions, we have isolated *Psychrobacter* spp. from the skin mucus of Atlantic salmon, which survived winter cold stress with and without ulcers.

Skin mucus samples were collected from Atlantic salmon raised in two flow-through systems (Huntsman Marine Science Center, HMSC; Saint Andrews Biological Station, SABS) by rolling sterile cotton swabs along and around the lateral line from behind the operculum to before the adipose and anal fins on a single side of salmon. Swabs from each group (with/without lesions) were pooled and placed immediately in 10 mL 25% glycerol in 50-mL Falcon tubes and kept at 4°C until bacterial isolation. Upon receipt of the samples, swabs in glycerol were vortexed for 30 s, and 100 µL were plated undiluted on Tryptic Soy Agar (TSA, MP Biomedicals, CA) supplemented with 2% NaCl cultured at 12°C aerobically for 3–5 days. A total of 19 single colonies were isolated from plates, and each colony was subcultured by streaking onto a new TSA plate supplemented with 2% NaCl cultured as above. Cells were harvested by rinsing each plate with 2 mL mixture of 25% glycerol and 0.05% peptone. Cell pellets were obtained from ca. 1.5 mL of the rinse by centrifugation at 7,500 × *g* for 10 min at 22°C. Total DNA was extracted from cell pellets using Qiagen’s DNeasy Blood & Tissue Kit following the manufacturer’s instructions (Qiagen, Germany) and subjected to whole genome sequencing (WGS) at Génome Québec. The libraries were constructed using the NEBNext Ultra II DNA Library Prep Kit (New England BioLabs), and a total of 775,027,371 150-bp paired-end reads were generated on the Illumina NovaSeq X Plus sequencing system. Data analysis was performed on the PHAC-NML’s high-performance computing cluster (Waffles), as in our previous study (4). Quality trimming was performed by fastp (version 0.23.2; [5]). Genome was assembled using SPAdes (version 3.15.5; (6)) with basic required option. Taxonomic classification was performed by GTDB-Tk (version 2.3.2 classify_wf; [7]). Annotation of the genome was carried out by NCBI’s PGAP using the default options (version 6.10; [8]).

An average of 40,790,914 reads were generated per strain with quality ≥ 39 (Table 1). The average genome size of these *Psychrobacter* spp. is 3.30 Mb with an average GC content of 43.7% (Table 1).

**TABLE 1:** Genome information of *Psychrobacter* spp. from Atlantic salmon

Strain	Raw reads	Genome size (Mb)	Gc (%)	Contigs	Scaffold N50 (kb)	Total genes	Protein-Coding genes	Assembly accession	Raw reads accession
1U1	35,036,000	3.3	42.5	18	512.3	2,722	2,649	GCA_051847535.1/	SRX29953798[accn]
1U2	33,202,834	4.6	43.5	1914	62.5	5,016	4,925	GCA_051847575.1/	SRX29953799[accn]
1Y1	39,299,061	3.5	43	488	89.5	3,224	3,133	GCA_051847395.1/	SRX29953795[accn]
1Y10	40,838,430	3.3	42.5	36	310.2	2,763	2,687	GCA_052259005.1/	SRX30164188[accn]
1Y11	38,504,105	3	44.5	38	242.4	2,533	2,474	GCA_052259045.1/	SRX30164180[accn]
1Y4	53,994,338	4.2	42.5	1242	181.4	4,270	4,177	GCA_051847555.1/	SRX29953796[accn]
1Y6	45,017,945	3.2	43	22	407.2	2,707	2,646	GCA_052258385.1/	SRX30164185[accn]
1Y7	41,105,650	3.2	43	29	416	2,730	2,669	GCA_052258505.1/	SRX30164186[accn]
1Y9	40,402,330	3.2	43	29	407.2	2,715	2,654	GCA_052258465.1/	SRX30164187[accn]
2U2	35,907,255	3	44.5	40	309.5	2,606	2,540	GCA_051847495.1/	SRX29953802[accn]
2U3	26,556,238	3.4	43.5	18	428.7	2,873	2,809	GCA_051847515.1/	SRX29953803[accn]
2Y4	37,385,253	3	44.5	39	228.5	2,607	2,540	GCA_051847435.1/	SRX29953800[accn]
2Y5	29,110,294	3	44.5	37	309.5	2,606	2,539	GCA_051847375.1/	SRX29953801[accn]
T6-1	48,772,284	3.2	44.5	852	8	3,263	3,196	GCA_052258445.1/	SRX30164178[accn]
T6-2	45,637,780	2.9	45	47	179.2	2,517	2,449	GCA_052259065.1/	SRX30164179[accn]
T6-3	46,845,884	2.9	45	38	185.2	2,509	2,440	GCA_052258165.1/	SRX30164181[accn]
T6-4	48,085,450	2.9	45	38	179.2	2,504	2,437	GCA_052258425.1/	SRX30164182[accn]
T6-5	43,729,669	3.4	43.5	31	333.8	2,920	2,846	GCA_052258485.1/	SRX30164183[accn]
T6-6	45,596,571	3.3	43.5	33	449.6	2,823	2,760	GCA_052258185.1/	SRX30164184[accn]

In conclusion, our study describes the commensal skin *Psychrobacter* spp. of Atlantic salmon raised in different environments and those that survived without the development of lesions.

## Data Availability

This Whole Genome Shotgun project has been deposited in Sequence Read Archive (SRA) under BioProject PRJNA1298876. The genome assembly and raw reads accession no. are listed in Table 1. The version described in this paper is the first version of each strain.
